# Exploring Chemical Transport through Food: A Proposal for a Comprehensive Approach to Predict Exposures

**DOI:** 10.1289/ehp.125-A26

**Published:** 2017-01-01

**Authors:** Nate Seltenrich

**Affiliations:** Nate Seltenrich covers science and the environment from Petaluma, CA. His work has appeared in *High Country News*, *Sierra*, *Yale Environment 360*, *Earth Island Journal*, and other regional and national publications.

The global industrial food system is emerging as a significant pathway through which people are exposed to potentially hazardous chemicals,^1^ but no method exists to assess the full extent to which chemicals are transported through food.^2^ Researchers Carla Ng of the University of Pittsburgh and Natalie von Goetz of ETH Zürich propose integrating an array of models and data sources into a comprehensive approach to better aid in risk assessment and exposure reduction.

**Figure:**
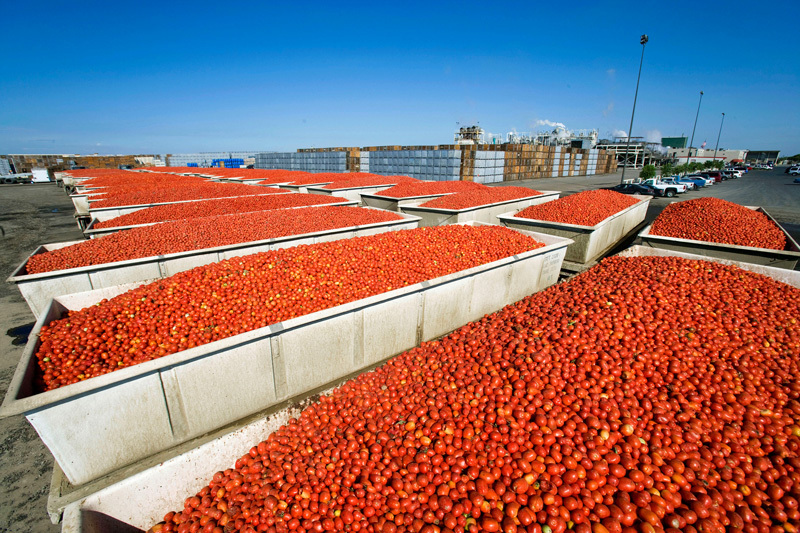
Food can pick up contaminants at multiple steps along the supply chain, from the point where raw ingredients are raised to the final dishing up of a meal. A new study explores a possible mechanism for anticipating and assessing chemical transport through food. © Andia/Alamy Stock Photo

Some commercial foods are contaminated with chemicals used in processing and packaging—examples include bisphenol A from jar lids, inks from cereal boxes, and plasticizers from hoses conveying beverages to bottles.^3^ ^,^ ^4^ Raw ingredients and fresh produce also can take on a chemical burden beginning with pesticides on the farm and industrial pollutants that accumulate in the environment, and continuing all the way through contamination during transport and distribution.^5^ ^,^ ^6^

Existing data and modeling capabilities address discrete steps in the pathway, such as chemical accumulation in local food webs,^7^ packaging,^4^ and human consumption.^8^ But while some chemicals are already monitored and regulated in foods by agencies such as the U.S. Food and Drug Administration^9^ ^,^ ^10^ and the European Food Safety Authority^11^ ^,^ ^12^—especially those intentionally added for taste, texture, appearance, or preservation—many more are not.

Non-intentionally added substances in foods are generally less well understood and regulated. These often originate within what Ng calls the biggest “black hole” in the global food-supply chain: processing, which can involve proprietary information. For example, the temperature to which final packaging is exposed during processing can affect levels of BPA transport, von Goetz says. Other steps along the way may impart impurities, by-products, contaminants from recycling processes, and breakdown products from additives and plastic polymers.^13^

“It’s a lot of confidential information, because industry is involved, but I would like it to be more known publicly,” von Goetz says. “If we know more about processing, we can know which chemicals to expect.”

Another way to gain insight into a food’s bill of health is to consider where it comes from. Limited country-of-origin labeling hampers efforts to understand the link between where a food is grown and the chemical burden it carries. However, data on trade flows, production, and consumption might be used to determine the likely country of origin.

Ng and von Goetz suggest that the notion of *terroir*, typically used to refer to the relationship between a food’s overall character and the environment in which it is produced, can also be applied to chemical transport. Local climate, pollutants in water and soil, and farming practices all play a role in determining a food’s chemical *terroir*.

“The residue level of specific pesticides depends on their use and therefore on both the crop and the regional pests, so that the *terroir* of the food is, to some extent, a predictor for the residue level of a specific pesticide and the human exposure to residues in consumed foods,” Ng and von Goetz write.

Hilko van der Voet, a biometrician at the Netherlands-based Wageningen University and Research Center, agrees with Ng and von Goetz that linking data about food origin, processing, and transport is an important goal. He suggests that a tiered approach to screening chemical exposures through food could help achieve this goal—that is, moving to advanced exposure modeling only after initial screening has indicated sufficient potential for hazardous exposures. But while reality will always be much more complex than any model can account for, van der Voet adds, it also is not necessary to model every detail of complex systems to obtain answers to risk assessment questions.
